# Enhancement of Tumor Cell Death by Combining *gef* Gene Mediated Therapy and New 1,4-Benzoxazepin-2,6-Dichloropurine Derivatives in Breast Cancer Cells

**DOI:** 10.3389/fphar.2018.00798

**Published:** 2018-07-26

**Authors:** Alberto Ramírez, Ana Conejo-García, Carmen Griñán-Lisón, Luisa C. López-Cara, Gema Jiménez, Joaquín M. Campos, Juan A. Marchal, Houria Boulaiz

**Affiliations:** ^1^Biopathology and Medicine Regenerative Institute, University of Granada, Granada, Spain; ^2^Biosanitary Institute of Granada, SAS-Universidad de Granada, Granada, Spain; ^3^Department of Pharmaceutical and Organic Chemistry, University of Granada, Granada, Spain; ^4^Excellence Research Unit “Modeling Nature” – Department of Human Anatomy and Embryology, University of Granada, Granada, Spain

**Keywords:** *gef* gene, 1, 4-benzoxazepin-2, 6-dichloropurine, breast cancer, combined therapy, gene therapy

## Abstract

New treatment modalities are urgently needed to better manage advanced breast cancer. Combination therapies are usually more effective than monotherapy. In this context, the use of cyclic and acyclic *O,N*-acetals derivative compounds in combination with the suicide *gef* gene shown a potent anti-tumor activity and represent a new generation of anticancer agents. Here, we evaluate the use of the *gef* gene to promote and increase the anti-tumor effect of cyclic and acyclic *O,N*-acetals purine derivatives and elucidate their mechanisms of action. Among all compounds tested, those with a nitro group and a cyclic pattern structures (FC-30b2, FC-29c, and bozepinib) are the most benefited from the *gef* gene effect. These compounds, in combination with *gef* gene, were able to abolish tumor cell proliferation with a minimal dose leading to more effective and less toxic chemotherapy. The effect of this combined therapy is triggered by apoptosis induction which can be found deregulated in the later stage of breast cancer. Moreover, the combined therapy leads to an increase of cell post-apoptotic secondary necrosis that is able to promote the immunogenicity of cancer cells leading to a successful treatment. This data suggests that this novel combination therapy represents a promising candidate for breast cancer treatment.

## Introduction

In accordance with the World Health Organization criteria, breast cancer is the malignancy with the highest incidence among women, with 30% of estimated new cases. Despite recent improvements in diagnosis and treatment, both incidence and prevalence are increasing, especially in industrialized countries. Thus, this malignancy is known to be the most important cause of cancer mortality among women, representing 14% of estimated all cancer deaths (Siegel et al., 2017). Conventional treatments (chemotherapy, radiotherapy, surgery, and hormone therapy) are efficient in early stages of the disease, however, they are only palliative for advanced breast cancer and have many side effects. Moreover, patients treated with current systemic therapies are known to suffer from multiple side effects (Malecki, 2012). These data uncover the demand to reduce the dose used in both chemotherapeutics and radiation treatment protocols below the most effective doses, or the withdrawal of a first-line treatment.

**FIGURE 1:**
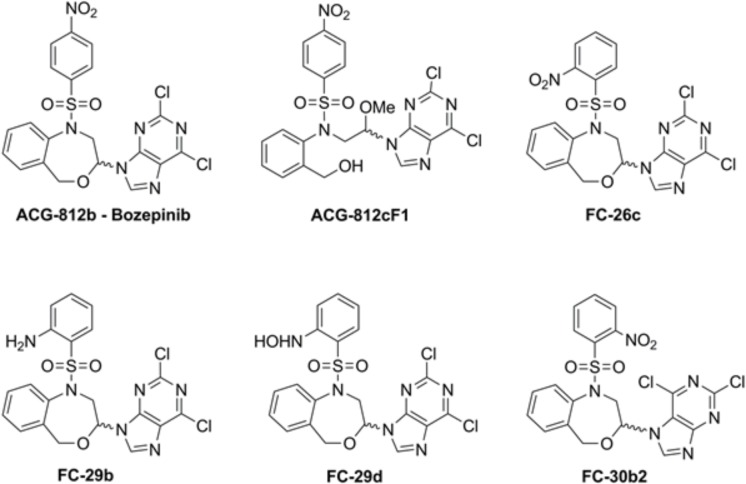
Chemical structure of the compounds.

Our previous studies highlighted the relevance of the antiproliferative activity of cyclic and acyclic *O,N-*acetals in either human breast and colon cancer cell lines in the micromolar range (Díaz-Gavilán et al., 2007; Conejo-García et al., 2008; Nuñez et al., 2008; López-Cara et al., 2011). Among them, (*R,S*)-2,6-dichloro-9-[1-(*p*-nitrobenzenesulfonyl)-1,2,3,5-tetrahydro-4,1-benzoxazepine-3-yl]-9*H*-purine (ACG-812b also called bozepinib, **Figure 1**) has shown to be the most potent and selective anti-tumor compound being able to leads apoptosis induction in breast and colon cancer cells mediated by the double-stranded RNA-dependent protein kinase (López-Cara et al., 2011; Marchal et al., 2013). Moreover, bozepinib inhibits the formation of both mamo- and colono-spheres and is able to abrogate the aldehyde dehydrogenase enriched (ALDH+) cancer stem cells (CSCs) subpopulations in the low micromolar range. CSCs represent a small tumor subpopulation known to be responsible for promoting and maintaining tumor growth by enhancing their self-renewal and differentiation capacity. They also play a key role in chemo and radiotherapy resistance due to their high metastatic potential and the ability to enter in a quiescent state (Visvader and Lindeman, 2008). The *in vivo* activity of Bozepinib was also shown trough the tumor and metastasis inhibition assessed in xenotransplanted nude mice without presenting sub-acute toxicity (Ramírez et al., 2014).

In addition, novel anti-tumor strategies like suicide gene therapy are attractive due to the failure of current treatment approaches and the chemoresistance to cure a high percentage of patients with advanced breast cancers. The mechanism in witch suicide gene therapy is based involves the delivery of a cytotoxic protein encoded by a gene into tumor cells (Amer, 2014). There are several suicide gene systems with proven anti-tumor efficacy (Navarro et al., 2016). With the goal to improve this therapy, our group has developed a novel and effective therapy strategy based on the use of *gef* gene. This gene belongs to a family with cell-killing functions in *E. coli*. The protein encoded by *gef* gene, a protein of 50 amino acids is anchored to the cytoplasmic membrane by the N-terminal portion and is able to induce cellular respiration arrest and cell death (Poulsen et al., 2005). In human tumor cells, *gef* gene has a potent anti-tumor effect by induction of cell cycle arrest and apoptosis (Boulaiz et al., 2003a,b) which could be used as a promising complementary strategy for the common treatment choices.

It is known that combination therapies are usually more effective than monotherapy. They can be used to achieve several important objectives that are less probable using monotherapy. Firstly, it provides an increase in cell death within an acceptable toxicity range for each drug, whenever that the dosage is not compromised and the tumor is sensitive to each medication; secondly, taking into account that the tumor is formed by a heterogeneous population, it increases the probability that some cells will respond in comparison with a single agent and finally, the use of a combined therapy may delay the apparition of drug resistance by triggering a rapid cell death and reducing the tumor mass (Dear et al., 2013). Currently, the combination of several systemic agents such as taxanes, aromatase inhibitors, monoclonal antibodies and capecitabine are used as a first-line treatment for metastatic breast cancer and, thus, appear to be associated with improved survival (Chia et al., 2007; Cardoso, 2016; Mansour et al., 2017). The successful use of these agents as first-and/or second-line treatments in clinical trials is reflected in current guideline recommendations to treat advanced breast cancer (Cardoso et al., 2017). However, in most cases, the combination of the classic chemotherapies leads to more side effects. Hence, the need to develop new therapeutic strategies capable of inhibiting, at very low doses, the proliferation of both quiescent and rapidly proliferating tumor cells to avoid recurrence and metastasis and improve the patient’s quality of life is imperative. With this goal and based on our experience using toxin gene-based therapy and the new synthesized cyclic and acyclic *O,N*-acetals, we developed a new strategy based on the combination of these therapeutic tools. The main goal of this work was to evaluate whether *gef* gene is able to enhance the anti-tumor effect of bozepinib and its derivatives ACG-812c, FC-26c, FC-29b, FC-29d, and FC-30b (**Figure 1**) and to explore the mechanisms involved in the effectiveness of this combination.

## Materials and Methods

### Cell Lines

The breast cancer cell line MCF-7 was kindly provided by Dr. N. Olea of the Sánchez Mora Tumoural Biology Institute (University Hospital of Granada). The MCF-7TG cell line, a *gef*-expressing breast cancer cell line controlled by a mouse mammary tumor virus promoter and inducible by dexamethasone (Dex) was derivates from MCF-7 cells following methodology previously described by us (Boulaiz et al., 2003b). A selected MCF-7pMAMneo empty vector positive clone was also used. Non-tumoral breast cell line MCF-10A (ATCC: CRL-10317) was provided by the Cell Bank of the University of Granada (Granada, Spain). MCF-7 cells were cultured in a humidified atmosphere at 37°C with 5% CO_2_ and Dulbecco’s modified Eagle Medium (DMEM) (Gibco, Grand Island, NY, United States) with 10% heat-inactivated fetal bovine serum (FBS) (Gibco), 2.7% sodium bicarbonate, 2% L-glutamine, 1% Hepes buffer, 500 mg/L ampicillin and 40 mg/L gentamicin. MCF-7TG cells were grown in the same medium described above supplemented by 200 μg/ml geneticin G418 and 1μM Dex to induce *gef* gene expression. MCF-10A grown in DMEM/F12 medium with 5% horse serum (HS), 0.02 μg/ml epithelial growth factor (EGF), 0.5 μg/ml hydrocortisone, 100 ng/ml cholera toxin and 0.01 μg/ml insulin.

### Drugs and Treatments

The cyclic and acyclic *O,N*-acetals purine derivatives were synthesized as previously described (López-Cara et al., 2011). The only acyclic structure is ACG-812cF1. The target compounds present the 2,6-dicholoropurine moiety linked by position 9 with the only exception of FC-30b2 where the purine is linked by position 7. The structures are substituted with *p*-nitrobenzenesulfonyl (bozepinib and ACG-812cF1), *o*-nitrobenzenesulfonyl (FC-26c, FC-30b2), *p*-hydroxylaminebenzenesulfonyl (FC-29d) and *p*-aminobenzenesulfonyl (FC-29b) groups (**Figure 1**).

DMSO was used to dissolve the compounds and after that they were aliquoted and stored at -20°C. Prior the experiment, each aliquot was diluted in medium to obtain the desired concentrations. The final DMSO concentration (used as a solvent) in cell culture was ≤0.1% v/v of DMSO. This concentration has no effect on cell proliferation. In each experiment we also use cells in medium with DMSO as controls.

### *In Vitro* Cytotoxicity Assays

To assess the effect of the different drugs on cell viability we used the sulforhodamine-B colorimetric assay. Cells were resuspended and seeded at a density of 5 × 10^3^ cells/well onto 12-well plates kept growing for 24 h. After that, the compounds were added at different concentrations to the cells (0.5, 0.7, 1, 2, 5, and 10 μM) (**Figure 1**). After 72 h treatment, the medium was discarded and fresh medium and treatment was added. Cells were maintained for 3 days more with the treatment. Finally, they were processed as previously described (Boulaiz et al., 2003b) using a Titertek Multiscan apparatus (Flow Laboratories, Irvine, United Kingdom) at 492 nm. We used the cell number for each cell stock before each cell growth experiment to set the linearity of the sulforhodamine-B assay. To calculate the IC_50_ values we used semilogarithmic dose–response curves to make a linear interpolation. All of the experiments were performed in triplicate wells for each treatment and were repeated at least twice.

### *In Vitro* Cell Proliferation Assays

MCF-7, MCF-7TG, and MCF-7pMAMneo cells were seeded at a density of 25 × 10^3^/well into six-well plates using the culture conditions above mentioned. After 24 h, fresh medium was added to the cells and treated with IC_50_ and 2^∗^IC_50_ of drugs cited in **Table 1**. No treatment was added to the control groups. To ensure the expression of the *gef* gene, MCF-7TG cells medium was always treated with 1 μM Dex. Cells in culture were always treated with or without drugs every 3 days when medium was changed up to the end of the assay. Four plates were used to run each treatment and time point (0, 3, 6, 9, 12, and 15 days of treatment). After the end of the treatment, we used sulforhodamine-B to stain the cells as previously described.

**Table 1:** Antiproliferative activities^a^ for cyclic and acyclic *O,N*-acetals compounds against the MCF-7 and MCF7TG cancer cell lines, and the epithelial MCF-10A cell line.

Compound	MCF-7 IC_50_ (μM)	MCF-7TG IC_50_ (μM)	MCF-10A IC_50_ (μM)
Bozepinib	1.232 ± 0.05	0.56 ± 0.01	6.33 ± 0.03
FC-26c	2.6 ± 0.10	0.65 ± 0.03	9.187 ± 0.08
ACG-812c-F1	9.43 ± 0.07	8.75 ± 0.18	17.48 ± 0.12
FC-29b	5.021 ± 0.12	1.9 ± 0.02	4.04 ± 0.09
FC-29d	8.98 ± 0.3	6.7 ± 0.01	47.12 ± 0.05
FC-30b2	7.75 ± 0.06	1.8 ± 0.08	9.9 ± 0.08

*^a^All experiments were conducted in duplicate and gave similar results. The data are means ± SEM of three independent determinations. The treatment time was 6 days.*

### Flow Cytometric Analysis of the Cell Cycle

Cells were grown at 70% confluence and treated with either DMSO alone or with different concentrations of the compounds corresponding to their IC_50_ values. Flow cytometry analysis was carried out after 72 h of treatment as described (Marchal et al., 2004). The results were representative of three independent experiments.

### Analysis of Apoptosis by Propidium Iodide and Annexin V-FITC Staining

Apoptosis was detected using the apoptosis detection kit for annexin V-FITC (Pharmingen, San Diego, CA, United States) by flow cytometry according to Boulaiz et al. (2003a). Both reagents annexin V-FITC and IP non-vital dye allowed the differentiation among: viable cells (no staining for both dyes), cells undergoing early apoptosis (positive for annexin V-FITC and negative for PI), cells undergoing late apoptosis (positive for both dyes), and necrotic cells (only showed IP staining). The results were representative of three independent experiments.

### Use of Confocal Microscopy for Apoptosis Detection

Labtek chamber-slide 8-well plates were used to seed the cells at a confluence of 5 × 10^3^ cells/well. After 24 h to allow cell adhesion, the treatment was added using the concentration needed for each compound. After aspirate culture medium, cells were washed using cold phosphate buffered saline (1X). Then we proceeded to incubation with both annexin V-FITC and propidium iodide (PI) for 15 min at room temperature in the dark. After that, cells were washed with binding buffer, preserved using mounting medium and cover slips before confocal microscopic imaging. Finally, cells were imaged by confocal microscopy using a Leica SP2 Confocal Microscope.

### Statistical Analysis

Data collected represent the mean ± standard deviation. Two-tailed Student *t*-test was used to compare differences between two groups. A two-tailed *p*-values < 0.05 was considered statistically significant.

## Results

### *gef* Gene Expression Enhances Cytotoxicity of Cyclic and Acyclic *O,N*-Acetals Compounds

**FIGURE 2:**
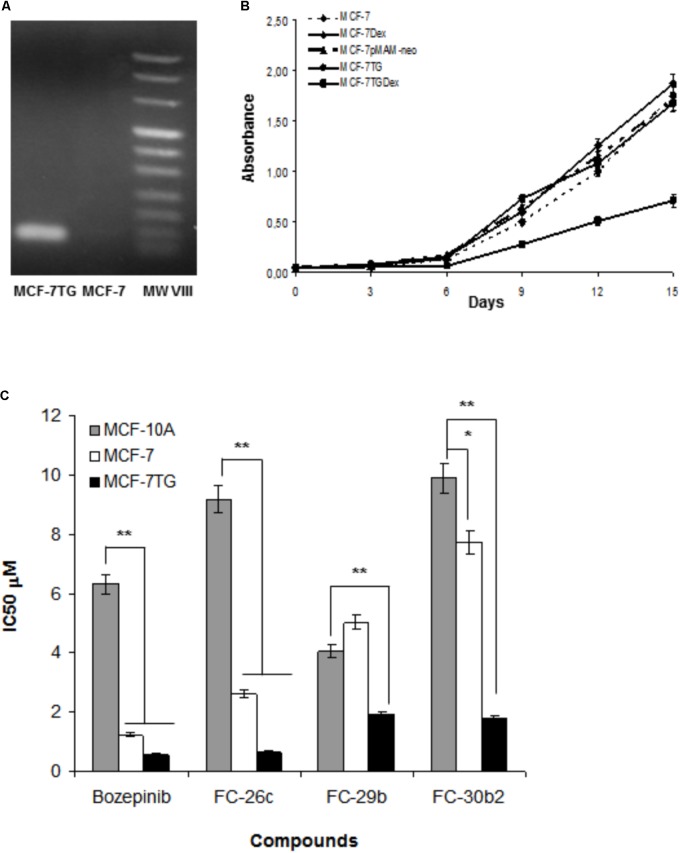
**(A)** Detection of the *gef* gene expression in MCF-7TG cells by RT-PCR. **(B)** Determination of the effects of *gef* gene expression on MCF-7 cell proliferation. MCF-7 Dex, MCF-7pMAM-neo, MCF-7TG were used as control. **(C)** Comparison between antiproliferative activities of bozepinib, FC-26c, FC-29b, and FC-30b2 against the MCF-7 and MCF-7TG cancer cell lines, and the MCF-10A epithelial cell line. Data are expressed as the mean ± standard error of the mean of three independent experiments. ^∗^*P* < 0.05, ^∗∗^*P* < 0.01 (*t*-test).

We have previously described the inhibition of cell proliferation due to *gef* gene activity in different cancer cell lines (Boulaiz et al., 2003a,b, 2008, 2014). In this work, we used a breast cancer cell line derived from the MCF-7 that stably expresses the *gef* gene after induction with 1 μM of dexamethasone. Firstly, we confirmed the expression of *gef* gene in MCF-7TG cells by RT-PCR (**Figure 2A**) and determine its anti-tumor effect by measuring the proliferation rate of MCF-7TG induced with 1 μM of dexamethasone during 15 days and comparing it with the MCF-7 parental cell, MCF-7 induced with 1 μM Dex (MCF-7Dex), MCF-7 transfected with *gef* without Dex induction (MCF-7T) and MCF-7 transfected with pMAMneo empty vector (MCF-7pMAMneo) used as controls (**Figure 2B**). Our results showed that MCF-7Dex, MCF-7pMAM-neo and MCF-7T showed a similar proliferation rate than MCF-7 cell line demonstrating that Dex and transfection process don’t have any antiproliferative effect on this cell line. However, in MCF-7TG we observed that *gef* gene induces an inhibition of the proliferation of 59.36 ± 2.9% compared to MCF-7.

Moreover, we have reported the potent anti-tumor effect of the novel cyclic and acyclic *O,N*-acetals purine derivatives: bozepinib, FC-26c, ACG-812c-F1, FC-29b, FC-29d, and FC-30b2 in a variety of cancer cell lines (López-Cara et al., 2011). In our study, we determined their inhibitory concentration (IC_50_) after 6 days of treatment in both MCF-7 and MCF-7TG cell lines. As shown in **Table 1**, the IC_50_ values for the different drugs were markedly reduced in the MCF-7TG cell line. This means that the *gef* gene expression sensitizes the cells to the different compounds; hence a much lower amount of the drugs is needed to induce the death of the same number of cells. Thus, the compounds that improved their antiproliferative effect in presence of the *gef* gene were FC-30b2, FC-26c, FC-29b, and bozepinib with 76.77%, 75%, 62.15%, and 54.55%, respectively (**Figure 2C**). These values confirm the potent anti-tumor effects resulting from the *gef* gene/drugs combination.

FC-29d and ACG-812c-F1 were benefited to a lesser extent from the *gef* gene expression with a decrease of the IC_50_ of 24.63% and 7.211%, respectively.

Moreover, a comparison between the two tumor cell lines (MCF-7 and MCF-7TG) and the MCF-10A epithelial-like cell line was established in order to define the selective activity of the compounds through the determination of the *in vitro* therapeutic index (TI) (**Table 2**). TI was better by far for almost all compounds in MCF-7TG than MCF-7 (**Table 2**). In addition, in MCF-7TG cells, the best TI was achieved by FC-26c, bozepinib, FC-29d, FC-30b2, and FC-29b (TIs = 14.13, 11.30, 7.032, and 5.5, 2.12, respectively). The ACG-812c-F1 did not show differences regarding TI with or without the *gef* gene expression (**Table 2**).

**Table 2:** Therapeutic indexes for cyclic and acyclic *O,N*-acetals compounds.

Compound N°	Therapeutic index (TI)	
	MCF-7	MCF-7TG
Bozepinib	5.14	11.30
FC-26c	3.53	14.13
ACG-812c-F1	1.85	1.99
FC-29b	0.8	2.12
FC-29d	5.24	7.032
FC-30b2	1.28	5.5

**FIGURE 3:**
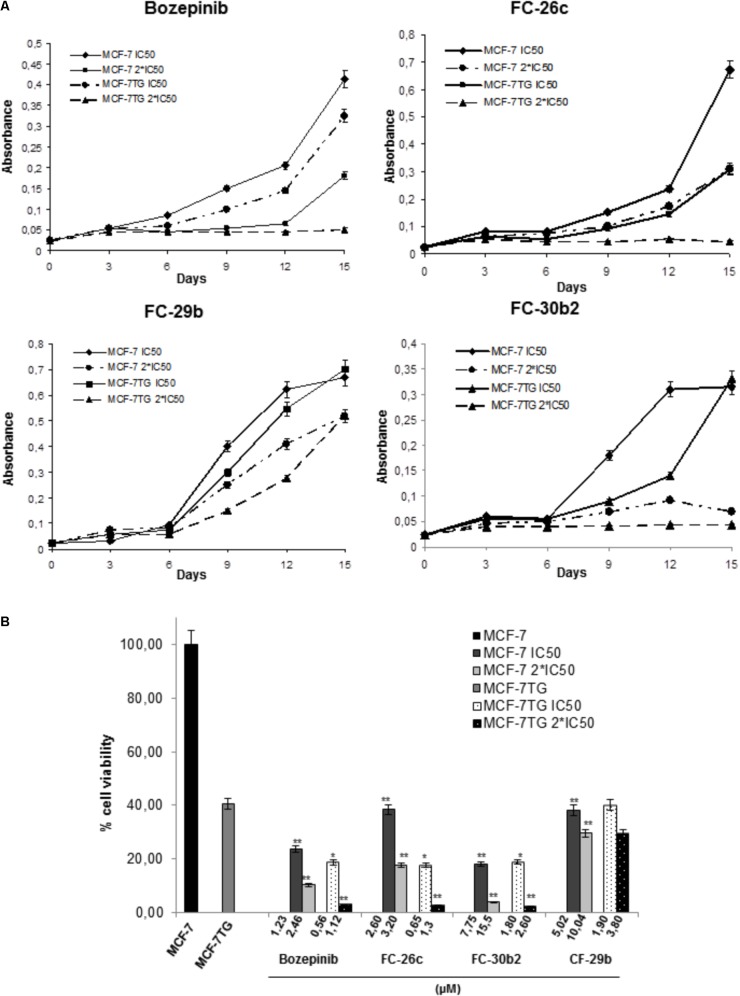
Determination of the effects of the combination of *gef* gene expression with bozepinib, FC-26c, FC-29b, and FC-30b2 on cell proliferation. **(A)** MCF-7 and MCF-7TG cell lines were treated with IC_50_ and 2^∗^IC_50_ of different compounds during 15 days. Data are expressed as the mean ± standard error of the mean of three independent experiments. **(B)** Percentage of cell viability after 15 days of treatment with different compounds expressed in comparison to the maximal MCF-7 (100%). The statistical analysis was performed using each cell line without drug exposure as controls. ^∗^*P* < 0.05, ^∗∗^*P* < 0.01 (*t*-test).

To test the antiproliferative action of the different compounds against MCF-7 and MCF-7TG cells over time, we measured the proliferation rate of these cell lines after being exposed to IC_50_ and double IC_50_ of the compounds (**Table 1**) during 3, 6, 9, 12, and 15 days of treatment (**Figure 3A**). Our results showed that until day 6 both MCF-7 and MCF-7TG cell lines shares a similar pattern growth, with a minimum proliferation rate in those exposed to IC_50_ and 2^∗^IC_50_ of different compounds. From day 6, differences between the cell line expressing the *gef* gene and the control cell line begin to be noticed, whether they are treated with the IC_50_ or with the double IC_50_ of the different drugs.

Thus, after 15 days of treatment by bozepinib in MCF-7 76% and 89.42% of growth inhibition using IC_50_ (1.23 μM) and 2^∗^IC_50_ (2.46 μM), respectively, was observed, while in the MCF-7TG cell line an inhibition of 81.42% and 97.14% using IC_50_ (0.56 μM) and 2^∗^IC_50_ (1.12 μM), respectively, has been achieved. The same trend was observed for FC-26c. After 15 days of treatment in MCF-7 an inhibition of 61.6% and 82,34% using IC_50_ (2.6 μM) and 2^∗^IC_50_ (3.2 μM), respectively, was reached, while in the MCF-7TG cell line we have achieved an inhibition of 82,34% and 97.43% using IC_50_ (0.65 μM) and 2^∗^IC_50_ (1.3 μM), respectively. In the same way, the FC-30b2 showed an inhibition of 82% and 96% using IC_50_ (7.75 μM) and 2^∗^IC_50_ (15.5), respectively, in MCF-7 cells, while in the MCF-7TG cell line we have achieved a growth inhibition of 81.14% and 97.57% using IC_50_ (1.8 μM) and 2^∗^IC_50_ (2.6 μM), respectively (**Figure 3B**). However, the FC-29b was the only compound that failed to induce a total inhibition of proliferation using 2^∗^IC_50_. In fact, without the *gef* gene expression, this compound showed an inhibition of 61.91% and 70.48% using IC_50_ (5.02 μM) and 2^∗^IC_50_ (10.04 μM), respectively. Similar percentages have been obtained using IC_50_ (1.9 μM) and 2^∗^IC_50_ (3.8 μM) in the presence of *gef* gene (60% and 70.48%, respectively) (**Figure 3B**).

These results indicate that the compounds have a synergistic effect with the *gef* gene but in a different way. When combined with *gef* gene the exposure to bozepinib, FC-26c and FC-30b2 has an enhanced synergetic effect while FC-29b has an additive synergetic effect.

### Apoptosis Is Involved in the Synergistic Effect of Combined Therapy

**FIGURE 4:**
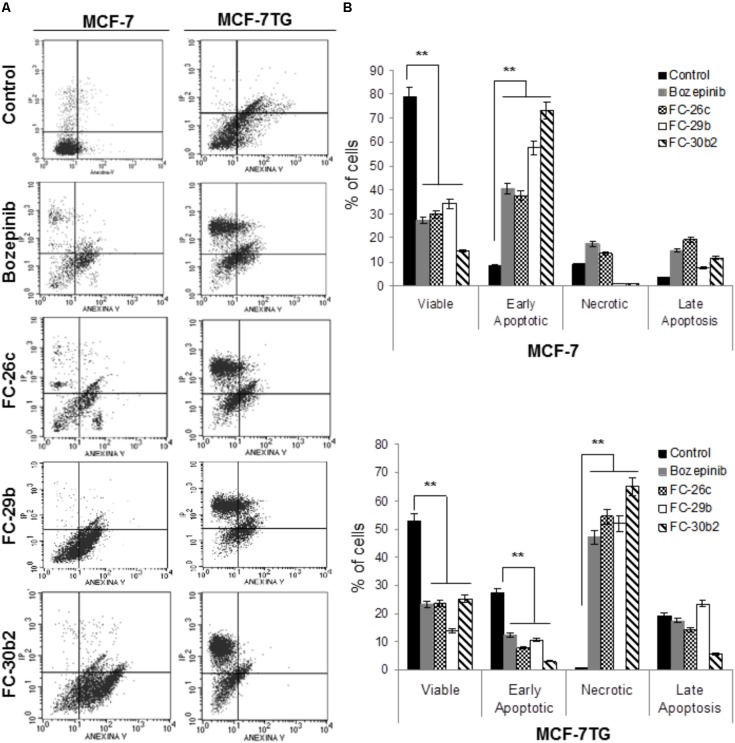
Cell death is enhanced by combination of *gef* gene and drugs. MCF-7 and MCF-7TG cell lines were mock-treated (control) or treated with IC_50_ of the most antiproliferative compounds for 72 h. Treated cells were then trypsinized and analyzed by flow cytometry using an annexin V-fluorescein isothiocyanate detection kit. **(A)** Representative images from flow cytometry analysis. **(B)** Data are expressed as the mean ± standard error of the mean of three independent experiments. ^∗∗^*P* < 0.01 (*t*-test).

Finally, in order to determine the role of apoptosis in the observed growth inhibition, we proceeded to use both flow cytometry and confocal microscopy. To carry out these assays, cells were treated with the IC_50_ values of the different compounds and stained using PI and annexin V after 72 h of drug treatment. MCF-7 and MCF-7TG non-treated cells were used as control (**Figure 4A**). In our MCF-7 control culture, 79.14 ± 3.95% of the cells were viable, 8.15 ± 0.40% were in early apoptosis, and 3.6 ± 0.18% undergoes late or final stages of apoptosis (*P* < 0.05). Interestingly, MCF-7 cell line treated for 72 h with the novel compounds suffered a significant increase in the subpopulation presenting early apoptosis in comparison to the control cells, showing percentages varying from 40.41 ± 2.02%, 37.63 ± 1.88%, 57.62 ± 2.88%, and 73.27 ± 3.66% for bozepinib, FC-26c, FC-29b, and FC-30b2, respectively. The late apoptotic cell subpopulation percentage was also increased in MCF-7 cell line treated with the drugs when compared to control cells (**Figure 4B**).

Moreover, in MCF-7TG cell line 52.89 ± 2.41% of the cells were viable, 27.14 ± 1.35% were in early apoptosis, 19.24 ± 1.15% were in the late stages of apoptosis and 0.74 ± 0.05% were necrotic. However, the combined expression of *gef* gene and different drugs induced a marked increase on necrotic cells (**Figure 4B**) varying from 47.03 ± 1.07%, 54.38 ± 2.3%, 51.92 ± 2.09%, 65.09 ± 2.47% for bozepinib, FC-26c, FC-29b, and FC-30b2 for each compound. Notably, the levels of necrotic cells induced by the combined therapy significantly increased in comparison with the corresponding drugs or *gef* gene alone.

**FIGURE 5:**
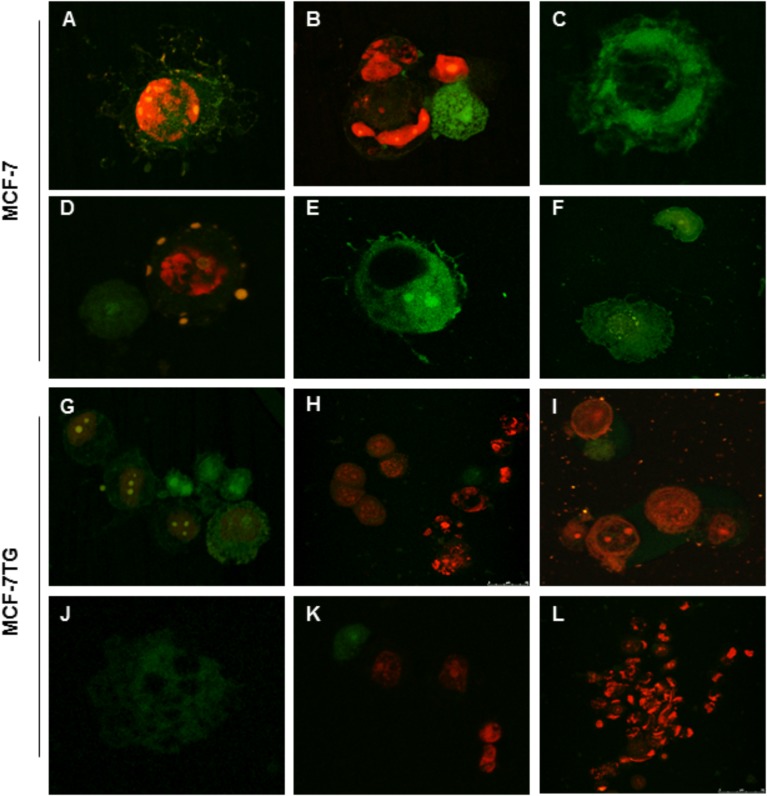
Confocal analysis of MCF-7 **(A)** and MCF-7TG **(G)** cells mock-treated and treated with bozepinib **(B,C,H,I)**, FC-26c **(D,J)**, FC-29b **(E,K)**, and FC-30b2 **(F,L)**. Cells were stained with annexin V-FITC and PI as described in the section “Materials and Methods.” Early apoptosis (stained positive for annexin V-FITC and negative for PI), late apoptosis or cell death (stained positive for both annexin V-FITC and PI) and necrosis (stained positive for PI) can be observed; viable cells are not visible. In MCF-7 treated cells we observe cells characteristic of late **(A,B,D)** and early apoptosis **(C,E,F)**. MCF-7TG cells treated with different drugs showed some early and late apoptotic cells **(G,H,J–L)** but much more necrotic cells **(H,I,K,L)**.

Confocal microscopy using FITC-conjugated annexin V and the nuclear non-vital dye PI was used to study the effects of compounds on the pattern of cell death. Early apoptosis, late apoptosis or cell death and necrosis can be observed; viable cells are not visible. In MCF-7 treated cells we observed signs of late apoptosis after the treatment revealed by the extrusion of sunspot-like apoptotic bodies (**Figures 5A,B,D**). We can also observe early apoptotic cells (**Figures 5C,E,F**). MCF-7TG cells treated with different drugs showed some early and late apoptotic cells (**Figures 5G,H,J,K**) but much more necrotic cells (**Figures 5H,I,L**).

### Combined *gef* Gene Expression and Drug Treatment Involves a G0/G1 Accumulation in MCF-7TG Cells

**FIGURE 6:**
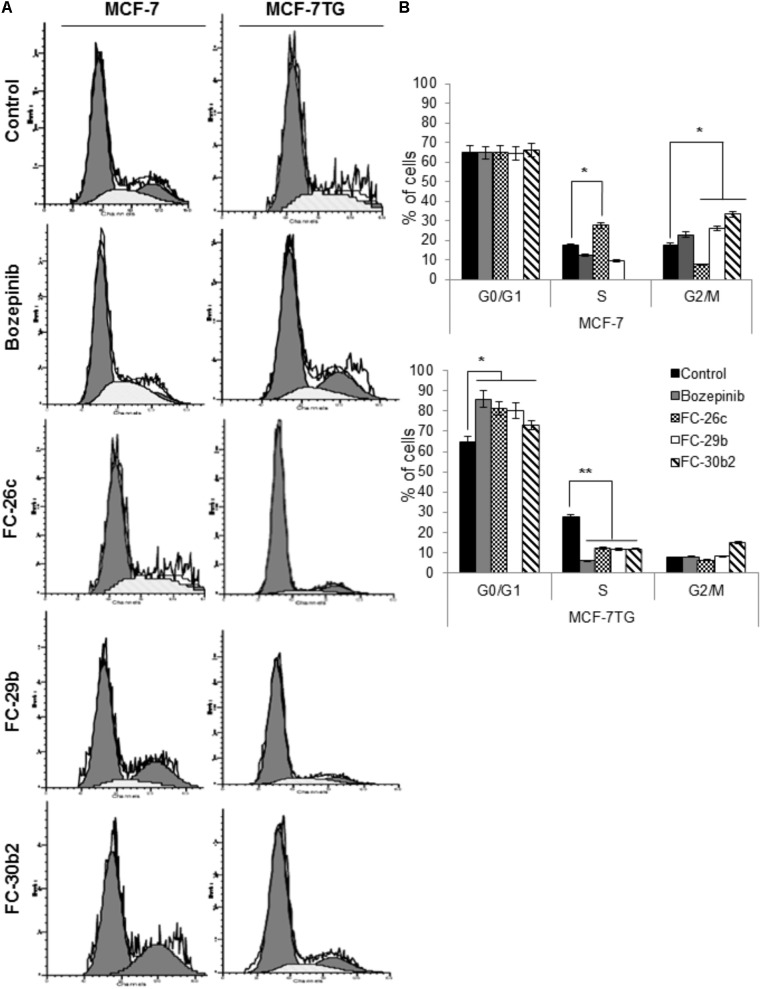
Combination of *gef* gene expression and drugs induce cell cycle arrest in G0/G1. MCF-7 and MCF-7TG cells were mock-treated (control) or treated with IC_50_ of bozepinib, FC-26c, FC-29b, and FC-30b2 for 72 h. Then, cells were trypsinized and analyzed by FACScan to distinguish populations of cells in different phases of the cycle as described in the section “Materials and Methods.” **(A)** Representative images from flow cytometry analysis. **(B)** Data are expressed as the mean ± standard error of the mean of three independent experiments. ^∗^*P* < 0.05, ^∗∗^*P* < 0.01 (*t*-test).

To study whether the antiproliferative effect of the combined treatment with compounds and *gef* gene involves changes in cell-cycle distribution, MCF-7 and MCF-7TG cell lines were treated using the IC_50_ of bozepinib, FC-26c, FC-29b, and FC-30b2 for 72 h and then analyzed by flow cytometry (**Figure 6A**). MCF-7 cell culture contained 65.03 ± 3.21% G0/G1 cells, 17.41 ± 0.81% S-phase cells and 17.56 ± 0.87% G2/M-phase cells. Interestingly, after *gef* gene induction for 72 h, MCF-7TG cell line suffered a gradual disappearance of G2/M (7.6 ± 0.28%) phase cells and they accumulate on S-phase (27.47 ± 1.34%). A similar behavior was observed after treatment of MCF-7 cell line with FC-26c while the other three drugs bozepinib, FC-29b and FC-30b2, caused an increase in the G2/M phase in the MCF-7 cell line (22.98 ± 1.14%, 26.12 ± 1.20%, and 33.22 ± 1.66%, respectively) and a decrease in S phase (12.29 ± 0.90%, 9.47 ± 0.30%, 0.63 ± 0.01%, respectively).

Nevertheless, the combined expression of *gef* gene and drugs induced a marked cell cycle arrest in G0/G1 phase being 85.98 ± 4.29%, 81.25 ± 4.05%, 80.07 ± 4.01%, and 72.96 ± 3.50% for bozepinib, FC-26c, FC-29b, and FC-30b2, respectively, and at the expense of cells in the S-phase and G2/M-phase population (**Figure 6B**).

## Discussion

Conventional cancer treatment strategies have been aimed at eradicating all cancer cells. Over the past 25 years, the systemic chemotherapy treatment of advanced breast cancer has suffered an evolution from the “anthracycline era” in the beginning of the 1980s, crossing the stage of the “taxane era” of the 1990s and finally establish in the “biological era” of the 2000s (Nabholtz et al., 2002). Standard chemotherapeutic approaches have relied on the maximum tolerated dose of cytotoxic drugs with a long off-therapy interval, leading to poor response, toxicity and eventual multidrug resistance (Kwak et al., 2017). In addition, the advances reached in the cancer development area have allowed researchers to identify two different populations inside tumors, a differentiated tumor cell subpopulation and an undifferentiated cell subpopulation or progenitor cells, also named CSCs. This heterogeneity of the tumor is responsible for the failure of most chemotherapeutic agents that eliminate only the cells that are in full division leaving quiescent cells such as the CSCs alive which in the long term results in recurrence and metastasis^8^. Thus, new treatment modalities combinations are urgently needed to better manage advanced breast cancer, including recurrent and metastatic cases (Lee and Djamgoz, 2017). In this context, we have previously described that on the one hand, different cyclic and acyclic *O,N*-acetals derivatives compounds and on the other hand, the suicide *gef* gene, shown potent anti-tumor activity and represent a new generation of anticancer agents (Boulaiz et al., 2003a,b, 2008, 2014; Nuñez et al., 2008; López-Cara et al., 2011; Ramírez et al., 2014). In this study, we have demonstrated that the combination of both therapeutic tools trigger the apparition of an adjuvant effect that enhances its anti-tumor activity in different breast cancer cell lines. Additionally, the *gef* gene sensitizes the breast cancer cells to the different compounds thus allowing decreasing its IC_50_ and increasing its TI.

It has been demonstrated that combination therapy enhances the efficacy of the therapy making possible to overcome the shortcomings of chemotherapy (Lee et al., 2010; Liu et al., 2011). The advances in the use of novel therapies combining cytotoxic drugs and gene therapy has been recently reported in different cancer types (Deharvengt et al., 2007; Abaza et al., 2008; Fandy et al., 2008; Lu et al., 2009; Touati et al., 2014; Zhang et al., 2015). In addition, several studies reported the ability of *gef* gene to improve the therapeutical effects of paclitaxel and doxorubicin in lung and breast cancer cells, respectively (Prados et al., 2008, 2010). In this work, we have selected bozepinib, FC-26c, ACG-812c-F1, FC-29b, FC-29d, and FC-30b2 as the six most potent and less toxic cyclic and acyclic *O-N-*acetals purine derivatives that we have previously synthesized in our group to analyze whether *gef* gene improves their therapeutic activity in breast cancer cells. Our results showed that combined therapy induces a decrease on IC_50_ values of all compounds in different degree. Thus, FC-30b2, FC-26c, FC-29b, and bozepinib mostly improved their antiproliferative effect in a remarkable way in the presence of *gef* gene with enhanced chemosensitivity up to 4.31-fold, 4-fold, 2.65-fold, and 2.2-fold, respectively. However, FC-29d and ACG-812c-F1 compounds have benefited to a lesser extent from the *gef* gene expression with only 1.08-fold and 1.34-fold enhanced chemosensitivity, respectively. Similar results were obtained by combination of *gef* with paclitaxel and docetaxel in MCF-7 breast cancer (Prados et al., 2010). The reason why ACG-812c-F1 and FC-29d were benefit lesser from the expression of the *gef* gene than the other compounds could be due to their chemical structures. These compounds present either an acyclic structure or the nitro group reduced to the hydroxylamino one, respectively. This seems to be detrimental to the benefit of combination therapy.

Moreover, *gef* gene greatly improved the TI of the different compounds. The ratio of the toxic dose to the therapeutic dose (*In vitro* TI = IC_50_ non-tumor cell line/IC_50_ tumor cell line) is defined as the *in vitro* TI of a drug (Kao et al., 2009). The best TI was achieved by FC-26c, bozepinib, FC-29d, FC-30b2, and FC-29b (TIs = 14.13, 11.30, 7.032, 5.5, and 2.12, respectively). The ACG-812c-F1 did not show significant differences regarding TI with or without the *gef* gene expression.

After the determination of the anti-tumor activity of the compounds against the different breast cell lines, we selected those that showed a balanced great cytotoxic effect and a better TI in the presence of *gef* gene, in order to determine its anti-tumor effect over time and its mechanism of action. Our results demonstrated that the expression of the *gef* gene combined with lower doses of the compounds caused a greater inhibition of cell proliferation compared to the control treated with high dose of drug only. Thus, until day 6 the same proliferation rate was observed with or without *gef* gene expression using IC_50_ and 2^∗^IC_50_ for each selected compound. From day 6, we observed that the compounds have a synergistic effect with the *gef* gene but in a different way. Combined with *gef* gene the exposure to bozepinib, FC-26c and FC-30b2, had an enhanced synergetic effect resulting in a total inhibition of proliferation using 2^∗^IC_50_ (1.12 μM, 1.3 μM, and 2.6 μM, respectively). While FC-29b, had an additive synergistic effect, it was only succeeded in inhibiting proliferation by 70% using 2^∗^IC_50_ in the presence (3.8 μM) or absence (10.04 μM) of *gef* gene. The biological data described here, support that the presence of the nitro group and the cyclic structures are the essential structural parameters for the synergistic effect with the *gef* gene of the tested compounds. We can find a similar case in the literature about an improvement of the cytotoxic activity related to the fusion gene CDglyTK. This gene encodes an enzyme with bifunctional activity in which are involved both CD- and TK-specific activities increasing the effectiveness, broad-spectral, and safe when compared to the use of the gene alone (Guowei et al., 2006). Moreover, in previous works we had shown that combination of *gef* with doxorubicin also decreased the viability of the MCF-7 cell line when compared to treatment alone. However, the effective doses (10 μM) were much higher than those necessary to induce a similar effect to that obtained by the FC-30b2, FC-29c, and bozepinib compounds (Prados et al., 2010). In addition, cell viability was reduced by 65.13% when *gef* and *apoptin* were synergistically co-expressed in colon DLD-1 treated cells after 10 days of treatment with doxycycline, while only the expression of *gef* or *apoptin* gene alone obtained a reduction of 35.9% and 47.95%, respectively (Boulaiz et al., 2014). The fact that the potentiation of the effect of combined therapy on cell proliferation is uncovered after a 6 days treatment was observed also after *gef* and *apoptin* genes co-expression and may be due of the needing for a target amount of gef protein in the tumor cells to trigger cell death (Boulaiz et al., 2014). The requirement of a critical concentration of gef protein to trigger cell death was reported in prokaryotic cells (Ronchel et al., 1998). These values confirm the potent anti-tumor effects resulting from combination of *gef* gene with very low doses of bozepinib, FC-26c, and FC-30b2 which has resulted in a total inhibition of proliferation after 15 days of treatment. Differences in the synergetic effect between FC-29b and the other compounds suggest the involvement of different signaling pathways as reported with other anti-tumor enantiomers (De Fátima et al., 2008).

It is known that cyclic and acyclic *O*,*N*-acetals purine derivatives can trigger apoptosis in tumor cells including breast and colon carcinoma (López-Cara et al., 2011; Marchal et al., 2013). Our group previously reported apoptosis induction due to the *gef* gene effect in breast (Boulaiz et al., 2003b, 2011) and colon (Ortiz et al., 2012) cancers. To clarify whether the combined effect of *gef* and bozepinib, FC-26c, FC-29b, and FC-30b2 compounds induces cytotoxicity by apoptosis and/or other mechanisms, cells with or without *gef* gene expression were treated with IC_50_ of different compounds and were stained with annexin V and PI. After that we performed both flow cytometry and confocal microscopy studies. These studies demonstrated that cytotoxicity due to the combined therapy was triggered by the induction of both apoptosis and cell death induced by a post-apoptotic secondary necrotic process. To assess the apoptosis induction we used the MCF-7 human breast cancer cell line. This cell line revealed to be very resistant where the demonstration of programmed cell death by known apoptosis-inducing agents has proven difficult and only few cytotoxic agents act preferentially through an apoptotic mechanism (Chadderton et al., 2000). In fact, at 72 h post-drug treatment with the novel compounds, MCF-7 cells were involved in an increase of early and late apoptotic cell subpopulations compared to the untreated control. However, the combined expression of *gef* gene and different drugs induced a marked increase on necrotic cells. This phenomenon was also observed after the co-expression of the *gef* and *apoptin* genes in colon cancer cells (Boulaiz et al., 2014). This necrosis should be classified as post-apoptotic secondary necrosis what is the natural outcome of the complete apoptotic program as described by Silva (2010). In fact, when the apoptotic cells are excessive *in vitro* and also when the phagocytic capacity of the organism is overwhelmed, the apoptotic cells progress to a secondary necrosis stage loosing progressively their structural integrity and they suffer of ballooning, permeabilization of the plasma membrane and finally they release cytoplasm contents (Sachet et al., 2017). However, it has been reported recently that the regulation of the secondary necrosis, rather than accidental (Rogers et al., 2017), constitutes a valuable target for the use of compounds with novel pharmacological activities aimed at enhance the immune response against cancer cell death (Galluzzi and Kroemer, 2017). The effects of the combined therapy to trigger cell death were also assessed by confocal microscopy after the use of FITC-conjugated annexin V and the nuclear non-vital PI stains. In the MCF-7 cell line treated with different compounds we observe cells characteristic of late apoptosis after the treatment. They can be seen in the form of sunspots the extrusion of apoptotic bodies (fragments of chromatin and cellular organelles, membrane coated). MCF-7TG cells treated with different drugs showed some early and late apoptotic cells but much more necrotic/secondary necrotic cells. In the cells used as controls, most of them showed negative staining for both dyes, except for a low number of cells showing the staining features of apoptosis. Our results supports the effect of the compounds over some stages of the apoptotic process where are described a series of morphological and biochemical types of cell death (Gooch and Yee, 1999; Marchal et al., 2004). Furthermore, our previous studies show that when used separately, both *gef* gene and bozepinib, one of the cyclic compounds that we analyzed in this study, are able to induce apoptosis through different pathways. It has been reported the association between the expression of the *gef gene* in the MCF-7 breast cancer cell line with an improvement in the prognosis and induction of apoptosis mediated by p53 signaling pathway (Boulaiz et al., 2011). However, our studies showed that bozepinib treatment had no effect on p53 and its induction was not necessary to trigger the apoptotic process in the breast and colon cancer cell lines under study (Ramírez et al., 2014). This could explain the enhanced synergetic effect obtained by combining both therapeutic tools.

The cell cycle is characterized by cell division and self renew. This process is subdivided in interphase (G1, S, and G2 phases) and mitosis, where the cell replicates the DNA and suffer a nuclear division (McIntosh, 2016; Maes et al., 2017). In the cell cycle progression from a phase to the next one are involved cell cycle proteins which regulates and coordinates the activity several checkpoint pathways. The abnormal activity of these cell cycle proteins and checkpoint pathways results in deregulation of cell cycle progression that is known to be one of the key hallmarks of cancer. Analysis of the cell cycle in MCF-7 showed that *gef* gene alone can induce the disappearance of cells undergoing G2/M phase leading to the accumulation of cells in the of S-phase. A similar behavior has been observed after treatment of MCF-7 cell line with FC-26c while the other three drugs bozepinib, FC-29b, and FC-30b2, caused an increase in the G2/M phase and a decrease in S phase. Nevertheless, the combined expression of *gef* gene and drugs induced a marked cell-cycle arrest in G0/G1 phase at the expense of cells in the S-phase and G2/M phase population. These results are consistent with the literature since many anticancer agents exerts its activity by arresting the cell cycle at the G0/G1, S and G2/M phase and finally they induce apoptosis (Murray, 2004; Kwan et al., 2016).

## Conclusion

In summary, to our knowledge, this is the first demonstration that *gef* gene mediated therapy synergizes with cyclic and acyclic *O,N*-acetals purine derivatives and enhance cell death in breast cancer cells. Within all the compounds that we have tested FC-30b2, FC-29c, and bozepinib are those that have most benefited from the gene *gef* expression being able to reach a total inhibition of tumor cell proliferation with a minimal dose, enhancing the effectiveness and decreasing the toxicity associated to chemotherapy. Thus, we have assessed how the presence of the nitro group and also the cyclic pattern of these structures affect to the synergistic effect with the *gef* gene.

This combined therapy is characterized by the induction of the apoptotic process which may be deficient in advanced or metastatic breast cancer. Moreover, the combined therapy increased cell post-apoptotic secondary necrosis, a promising target for the development of novel 1,4-benzoxazepin-2,6-dichloropurine drugs that may enhance the immunogenicity of cancer cells leading to successful new treatments. However, further studies are necessary to establish the mechanism of action of this combined therapy and its usefulness *in vivo*.

## Author Contributions

AR conducted the experiments, data analysis, and interpretation. AC-G and CG-L synthesis of compounds and interpretation of results. JC and GJ data analysis and interpretation, and synthesis of compounds. JM design of the study, data analysis, and article review. HB conception and design of the study, data analysis and interpretation, drafting the article, and final approval of submitted manuscript. All authors final approval of submitted manuscript.

## Conflict of Interest Statement

The authors declare that the research was conducted in the absence of any commercial or financial relationships that could be construed as a potential conflict of interest.
